# Facilitators and Barriers to Implementing the 4Ms Framework of Age-Friendly Health Systems: A Scoping Review

**DOI:** 10.3390/nursrep14020070

**Published:** 2024-04-15

**Authors:** Huey-Ming Tzeng, Hannah E. Franks, Elise Passy

**Affiliations:** 1 School of Nursing, The University of Texas Medical Branch at Galveston, Galveston, TX 77555-1132, USA; 2 School of Health Professions, The University of Texas Medical Branch at Galveston, Galveston, TX 77555-1132, USA; hefranks@utmb.edu; 3 Alzheimer’s Health Systems Director-Texas, National Division of Health Systems, Alzheimer’s Association, Houston, TX 77087, USA; epassy@alz.org

**Keywords:** aged, Medicare, delivery of healthcare

## Abstract

Background: This scoping review explored the evidence in the peer-reviewed published journal literature to identify the facilitators and barriers to implementing the 4Ms Framework of Age-Friendly Health Systems in inpatient and outpatient clinical settings. Methods: Our search strategy focused on primary and secondary data sources that described the barriers and facilitators of incorporating the 4Ms Framework in clinical settings. We focused on older adults 65 years and older and followed the guidelines of the Preferred Reporting Items for Systematic Reviews and Meta-Analyses Extension for Scoping Reviews (PRISMA-SCR). Results: The evidence analyses of the 19 identified articles revealed six facilitator themes and five barrier themes to implementing the 4Ms Framework of Age-Friendly Health Systems in inpatient and outpatient clinical settings. The most recurring facilitator theme was embedding the 4Ms Framework into routine clinical practice with clinical pathways and designated personnel. The most frequently reported barrier theme was the lack of clinicians’ buy-in. Conclusions: Future research may translate the findings of this scoping review into a facilitator and barrier checklist or a “reality-check” measure to monitor the progress of the journey of embracing the 4Ms Framework in outpatient or inpatient clinical settings. This study was not registered.

## 1. Introduction

The Institute for Healthcare Improvement’s (IHI) 4Ms Framework of Age-Friendly Health Systems includes four components: (1) knowing “What Matters” to each person; (2) preventing, identifying, treating, and managing “mentation” issues; (3) supporting “mobility” needs; and (4) necessary “medication” [1,2]. Despite the benefits of age-friendly health systems on older adults’ health outcomes, there is limited evidence on improving health-system-level and community-level metrics to support the implementation and sustainability of age-friendly health systems [3]. A recent interview study [4] explored the insights of adopting the 4Ms Framework in three health systems that were early adopters of the Framework and found that the common barriers to implementing the IHI’s 4Ms Framework included disengaged physicians, siloed implementation efforts (which led to problems with collaborations and scaling), and challenges in implementing “What Matters” in a meaningful way during clinical encounters [4]. A frontline culture change to sustain the 4Ms implementation is warranted. Successful efforts are dependent upon effective top-down communication, redesign of the healthcare system’s infrastructure to provide effective and tailored care to older adults, and provision of clinical education and support [4]. To our knowledge, no scoping review studies have examined the facilitators and barriers when implementing the IHI’s 4Ms Framework. In light of the existing knowledge gaps [3,4], the Alzheimer’s Association (AA) recommended the adoption of the IHI’s 4Ms Framework of Age-Friendly Health Systems; however, health systems and clinics need evidence on how the implementation of the IHI’s 4Ms Framework has better supported the health and well-being of people aged 65 years and older by providing older-adult-friendly clinical encounters (e.g., offering Medicare beneficiaries free annual wellness visits to access preventive services in ways that make sense to older adults) [5,6,7].

This scoping review explored the evidence in the peer-reviewed journal literature to identify the facilitators and barriers to implementing the 4Ms Framework of Age-Friendly Health Systems. The overarching research question was: What are the facilitators and barriers to implementing the 4Ms Framework of Age-Friendly Health Systems in inpatient and outpatient clinical settings?

## 2. Materials and Methods

Our search strategy focused on primary and secondary data sources (including systematic reviews) that described the barriers and facilitators related to incorporating the 4Ms Framework in hospital and outpatient clinical settings. We focused on older adults, defined by the researchers as adults aged 65 years and older. We followed the guidelines of the Preferred Reporting Items for Systematic Reviews and Meta-Analyses Extension for Scoping Reviews (PRISMA-SCR) [8].

This study was not registered. No published or registered protocol was in place before the study commenced.

Articles were included if they met the following criteria: (1) were peer-reviewed, (2) reported implementation of the 4Ms Framework of Age-Friendly Health Systems, (3) included hospitals or primary care clinics caring for older adults, and (4) were published on or before 10 August 2023 and were written in English.

We worked with a librarian to identify two conceptual groups of combined and individually adapted terms for each database-specific search. These terms included “4Ms” and “Age-Friendly Health Systems”. Table 1 provides the search syntax generated and modified for each electronic database. Studies were identified by searching these databases and hand-searching reference lists of the included articles.

The following databases were searched: MEDLINE–Ovid and EBSCOhost. The initial search was performed between 8 September 2022 and 13 December 2022, with five updated searches on 17 March 2023, 28 April 2023, 20 June 2023, 10 August 2023, and 10 January 2024. We hand-searched references of the included articles and used the snowball method to identify relevant papers. No gray literature was included in the complementary searches.

As for the selection of evidence sources, the first two authors screened the citations and articles against the preset inclusion criteria described in the “eligibility criteria” section. We applied the same approach to identify articles for inclusion in the initial and updated searches. First, we screened the titles and abstracts of all retrieved articles from the library databases and removed the duplicates. Next, we retrieved the remaining articles’ full texts and reviewed them for relevance according to the research question, assigning a score of either 0 (not relevant) or 1 (relevant). We then discussed conflicts and discrepancies between interrater scores to resolve them. The overall interrater reliability Kappa score was calculated to be 0.708 (standard error = 0.089, *p* < 0.001) using the Statistical Package for the Social Sciences (SPSS) [9]. All citations were imported or manually entered using Endnote X9 reference manager [10].

We extracted the following preidentified data from the final included articles: author names, title and date of publication, study type, design, data collection methods, study setting, sample size and description, outcome measures used, findings, and facilitators and barriers to the implementation of the 4Ms Framework. For each selected study, the first two authors extracted and coded the data for facilitators and barriers as described in each included article. All data were compiled into a table using Microsoft^®^ Word Version 16.28 for Mac [11].

We appraised each included article’s characteristics and methodological quality using the JBI critical appraisal tool for quantitative studies (e.g., randomized clinical, prospective, retrospective, and cross-sectional studies) [12]. The JBI critical appraisal tool evaluates the rigor, trustworthiness, relevance, and potential for bias in study designs, conduct, and analysis [12]. See Supplementary Materials Tables S1–S5 for the critical appraisal data of the included studies using the JBI critical appraisal tools for study designs.

We analyzed the findings from the included articles to identify the barriers and facilitators of implementing the 4Ms Framework. The first two authors met weekly via the Zoom online meeting site to review codes and themes from the data analyses. Conflicting themes were resolved by discussion.

### 2.1. Selection of Evidence Sources

**Figure 1:**
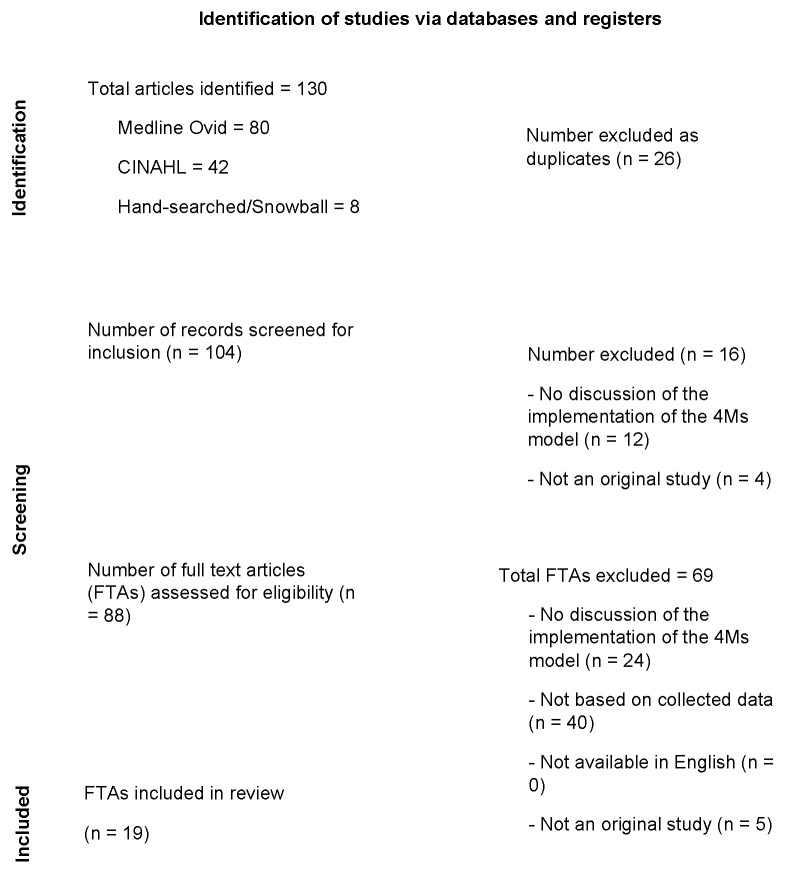
Flowchart of identification of studies via databases.

We identified 130 articles from the two databases (n = 122) and by the hand-searching/snowball method (n = 8). Of these 130 articles, 26 were duplicates, resulting in 104 articles to be further screened. After screening the title and abstracts, we excluded 16 articles, leaving 88 for which we retrieved and assessed the full texts for eligibility. After screening the full texts, we excluded 69 articles, leaving 19 for data extraction and final review. Full-text articles were excluded from final screening for the following reasons: (1) no discussion of the implementation of the 4Ms Framework, (2) not based on collected data, (3) the article was not written in English, or (4) the article was not an original study (i.e., discussion paper, editorial, commentary, essay, or dissertation) (Figure 1).

### 2.2. Characteristics of Evidence Sources

Table 2 summarizes the general methodological characteristics of the 19 included articles. Four were published in 2020, three in 2021, six in 2022, and six in 2023. Seven (36.8%) were quantitative studies. Two (10.5%) were qualitative studies. Two (10.5%) used a mixed methods approach. Two (10.5%) other articles were meta-analyses, scoping, or integrative reviews. Five (26.3%) used a cross-sectional design, four (21.1%) were prospective studies, five (26.3%) were retrospective studies, and seven (36.8%) were quality improvement projects. Seven (36.8%) studies used surveys, two (10.5%) studies used observation, four (21.1%) studies used interviews, two (10.5%) studies used focus groups, two (10.5%) conducted scoping reviews, eight (42.1%) studies used retrospective data collection methods, one (5.3%) used organizational and stakeholder assessment, two (10.5%) used physical assessments, and one (5.3%) used workflow mapping. There were six different settings in which these studies took place: five (26.3%) in inpatient hospitals, two (10.5%) in emergency departments, one (5.3%) in an independent residential community and community organization (e.g., faith-based organizations, libraries, healthcare organizations, activity centers), two (10.5%) in rural healthcare systems, ten (52.6%) in outpatient clinics, and one (5.3%) that was not specified.

### 2.3. Synthesis of Results

As for the content analysis and the process of data charting, we extracted data from the final selected articles based on preidentified data items: author(s), title and date of publication, study type and design, materials and methodology, data collection methods, stage of the care continuum on which the study focused, setting, barriers or facilitators, limitations, and lessons learned. Table 3 includes the summary of the evidence of the included studies (Table 3 can be found after the references due to the length of the table).

Based on the summary of the evidence in Table 3, we used the 4Ms Framework of Age-Friendly Health Systems to guide the synthesis of results and to organize the findings (i.e., the descriptive content analysis and descriptive results of the review synthesis). In other words, the first two authors analyzed the findings in Table 3 to identify the barriers and facilitators of implementing the 4Ms Framework. For each selected study, the first two authors extracted and coded the data as barriers and facilitators of implementing the 4Ms Framework. The main themes were then developed based on the identified codes (subthemes) related to barriers and facilitators (as shown in Table 4 and Table 5). All data were compiled into a table or spreadsheet using Microsoft^®^ Word Version 16.28 for Mac [11] for manuscript preparation purposes. The first two authors met weekly to review and refine codes and themes from the data analyses, and conflicting themes were resolved by discussion during the weekly meetings. Table 4 describes the facilitator themes and related subthemes to implementing the 4Ms Framework. Table 5 presents the barrier themes and associated subthemes to implementing the 4Ms Framework as reported in all included studies.

As part of the content analysis, we carefully recorded each identified code (i.e., quantifying the observed codes as yes = present, no = not present) using Microsoft Excel. We matched the identified codes (subthemes) with the corresponding themes. Then, we generated the frequency of each main theme and summarized the findings in Table 6. Each article may be cited to more than one facilitator or barrier theme. In other words, Table 6 includes only the main themes of the identified facilitator and barrier themes (i.e., the number of included articles for each identified theme), as stated in Table 4 and Table 5.

#### 2.3.1. Facilitators

As shown in Table 4, we identified the following themes as facilitators to implementing the 4Ms Framework: (1) frequency of patient participation in age-friendly care, (2) aligning the health system’s mission with the 4Ms Framework, (3) infrastructure readiness to embrace the 4Ms Framework, and (4) and embedding the 4Ms Framework into routine clinical practice with clinical pathways and designated personnel. Table 6 summarizes that the most frequently mentioned facilitator theme was embedding the 4Ms Framework into routine clinical practice with clinical pathways and designated personnel (11 articles, 57.9%), followed by infrastructure readiness to embrace the 4Ms Framework (9 articles, 47.4%).

The facilitator theme of embedding the 4Ms Framework into routine clinical practice with clinical pathways and designated personnel included six subthemes: (1) adopting geriatric syndrome screening before introducing interventions; (2) alignment between the entire 4Ms Framework or part of the 4Ms Framework and clinical practice; (3) alignment between the 4Ms Framework component of “What Matters?” and patients’ interests in improving health; (4) alignment between the 4Ms Framework component of mentation and clinical practice; (5) alignment between the 4Ms Framework component of medication and clinical practice; and (6) alignment between the 4Ms Framework component of mobility and clinical practice. Within this facilitator theme, the most often recurring subthemes were the alignment between the 4Ms Framework component of “What Matters?” and patients’ interests in improving health (e.g., giving older adults a choice [18]; discussions with patients regarding goals, preferences, priorities, their knowledge about their situation, and what brings the patient comfort during difficult moments [27]; adding facilitating questions regarding “What Matters” to intake paperwork [28]; having designated healthcare providers and processes (through combined home and telehealth visits within geriatric emergency departments [EDs]) to address “What Matters” and identify unmet care needs [29]; and incorporating a “What Matters” conversation guide tailored for ED settings to ascertain fears or concerns about the patient’s healthcare needs and identify the outcomes patients most want [24]) (Table 4).

#### 2.3.2. Barriers

Table 5 shows the identified five barrier themes on the implementation of the 4Ms Framework, which were (1) patients unable to actively participate in age-friendly care; (2) lack of infrastructure readiness to embrace the 4Ms Framework in clinical practice; (3) lack of clinicians’ buy-in; (4) challenges in incorporating the 4Ms components in clinical practice; and (5) lack of clinician awareness. As shown in Table 6, the most frequently reported barrier theme was the lack of clinician’s buy-in (eight articles, 42.1%), which included six subthemes: (1) clinicians’ concerns about adding extra burden and steps during clinic visits; (2) lack of time to learn about the 4Ms Framework; (3) limited time to implement the 4Ms Framework; (4) lack of full buy-in from clinicians and health systems in eliciting older adults’ goals and values (i.e., “What Matters?”); (5) limited published literature on adoption of the 4Ms Framework; and (6) siloed implementation efforts across settings within a health system leading to limited synergies and scaling of the 4Ms Framework. Within this barrier theme, the most common barrier subtheme was limited time to implement the 4Ms Framework (e.g., limited time available for implementation of the 4Ms [18,25]; limited time available to participate in the monthly grand round [13]; and limited available time during medical visits [23]) (Table 5).

## 3. Discussion

In this scoping review, we explored the evidence in 19 data-based, peer-reviewed journal articles to identify facilitators and barriers to implementing the 4Ms Framework of Age-Friendly Health Systems in inpatient and outpatient clinical settings. We identified four facilitator themes for implementing the 4Ms Framework: (1) frequency of patient participation in age-friendly care, (2) aligning the health system’s mission with the 4Ms Framework, (3) readiness of health system infrastructure to implement the 4Ms Framework, and (4) and embedding the 4Ms Framework into routine clinical practice with clinical pathways and designated personnel, as the most frequently mentioned facilitator theme. These facilitators were geared toward health-system-level policy and technological and personnel infrastructure readiness. These findings were consistent with previous research [4] that implementation success required redesigning the healthcare system infrastructure along with needed clinical education and support [4]

We also identified five barrier themes: (1) patients unable to actively participate in age-friendly care; (2) lack of infrastructure readiness to embrace the 4Ms Framework in clinical practice; (3) lack of clinicians’ buy-in, as the most frequently mentioned barrier theme; (4) challenges in incorporating the 4Ms components in clinical practice; and (5) lack of clinician awareness. Our findings were consistent with previous interview studies [4] that identified the common barriers to implementing the IHI’s 4Ms Framework as mostly related to clinicians’ attitudes toward the benefits of adopting the 4Ms Framework (i.e., disengaged physicians) [4]

In summary, our findings revealed many significant system factors (e.g., infrastructure readiness) that may hinder or support the implementation of the 4Ms Framework of Age-Friendly Health Systems [4,13,14,15,16,17,18,21,22,23,24,25,26,27,28,29,30]. As an example, a recent review [33] around the implementation of the streamlining framework (for streamlining cancer multidisciplinary meetings) in the United Kingdom’s national health system identified similar system issues. This review suggested several strategies to overcome challenges to its implementation (e.g., securing buy-in from key clinician and administration stakeholders, desiring clearly defined management approaches that include triage, assessment of cancer case complexity, the roles of clinicians and clinical staff, and acknowledging that the standard of care cannot be universally applied without the consideration of the variations across hospitals and clinics) [33]. Another review [34] summarized the enablers of chronic disease prevention and management for the Aboriginal people in Australia (e.g., culturally acceptable and safe services, patient–provider partnerships, primary healthcare service attributes, and clinical care pathways). This review emphasized the need to enable place-based partnerships across patients, providers, and policymakers to develop strategies that align with local community priorities as another system factor [34]. Further in-depth research is warranted to comprehend these identified system issues of implementing the 4Ms Framework. It is critical to link these identified system issues to a broader health systems literature and what that means for the ongoing development and implementation of the 4Ms Framework (e.g., using implementation science frameworks to guide review studies and studies that collect primary data [33]).

### 3.1. Practical Implications

The findings of this scoping review could be translated into a facilitator and barrier checklist or a “reality-check” measure to monitor the journey of embracing the 4Ms Framework in outpatient or inpatient clinical settings. Strategies may be developed to address the barriers and leverage those facilitators unique to each clinical setting (e.g., its infrastructure readiness [4,13,15,16,18,25,26,27,28], clinical practice and staffing patterns [14,16,17,18,21,23,24,25,27,28,29]). Clinicians’ buy-in is another system improvement aspect that desires health system executives’ and administrators’ attention before implementing the 4Ms Framework of Age-Friendly Health Systems [4,13,18,22,23,25,28,30].

### 3.2. Study Strengths and Limitations

The main strength of this scoping review was summarizing the facilitators and barriers of implementing the relatively new IHI 4Ms Framework in a hospital or outpatient clinic setting. As a limitation of this scoping review, we excluded the studies that focused on healthcare providers’ education or training-related interventions. This exclusion narrowed the scope of this review to a focus on implementation-related matters in clinical settings.

## 4. Conclusions

The evidence analyses of the 19 original peer-reviewed articles revealed a total of six facilitator themes and five barrier themes to implementing the 4Ms Framework of Age-Friendly Health Systems in inpatient and outpatient clinical settings. The most frequent recurring facilitator theme was embedding the 4Ms Framework into routine clinical practice with clinical pathways and designated personnel [18,24,27,28,29]. The most frequently reported barrier theme was the lack of clinician buy-in [13,18,23,25]. Future research may translate the findings of this scoping review into a facilitator and barrier checklist or a “reality-check” scale to monitor the progress of the journey of embracing the 4Ms Framework in outpatient or inpatient clinical settings.

## Figures and Tables

**Table 1:** Keyword search syntax and search strategy for the two library databases (Ovid–MEDLINE and EBSCOHost–CINAHL databases).

1. 4Ms
2. “Age-Friendly Health Systems”
3. 4 M Framework
4. 1 or 2 or 3
5. Limit 4 to (English language and “all aged (65 and over)”)

Notes: This search strategy was put together with the help of a professional librarian to ensure a comprehensive search of keywords and MeSH terms.

**Table 2:** Summary of the included studies by types.

		n (%)	Article Citations
Publication Year	2020	4 (21.1)	[13,14,15,16]
	2021	3 (15.8)	[17,18,19]
	2022	6 (31.6)	[20,21,22,23,24,25]
	2023	6 (31.6)	[13,26,27,28,29,30]
Study Type	Quantitative	11 (57.9)	[13,16,17,20,21,23,25,26,27,28,29]
	Qualitative	4 (21.1)	[4,13,18,22,24]
	Mixed methods (including both qualitative and quantitative data collections)	2 (10.5)	[15,19]
	Meta-analysis, scoping review, or integrative review	2 (10.5)	[14,30]
Study Design	Cross-sectional	5 (26.3)	[4,13,19,23,24]
	Prospective	4 (21.1)	[16,25,26,31]
	Retrospective	5 (26.3)	[17,20,21,27,28]
	Quality improvement project	7 (36.8)	[15,16,18,21,25,28]
Data Collection Methods	Survey	7 (36.8)	[13,15,18,19,22,23,29]
	Observation	2 (10.5)	[18,22]
	Interview	4 (21.1)	[4,18,22,24]
	Focus groups	2 (10.5)	[18,22]
	Peer-reviewed journal articles	2 (10.5)	[14,30]
	Retrospective data collection (e.g., administrative record or electronic health record)	8 (42.1)	[15,16,20,21,26,27,28,29]
	Assessment (e.g., organizational readiness and stakeholder assessment)	1 (5.3)	[18]
	Physical assessment (e.g., Timed Up and Go)	2 (10.5)	[17,25]
	Workflow mapping	1 (5.3)	[18]
Country of the Study Sites	Australia	1 (5.3)	[14]
	Multiple European countries	1 (5.3)	[30]
	United States	18 (94.7)	[4,13,15,16,17,18,19,20,21,23,24,25,26,27,28,29,30,32]
Setting	Inpatient hospital (e.g., acute care, orthopedics unit, consultative geriatric care)	5 (26.3)	[4,13,21,27,28]
	Emergency department	2 (10.5)	[24,29]
	Independent residential communities and community organizations (e.g., faith-based organizations, libraries, healthcare organizations, activity centers)	1 (5.3)	[25]
	Rural healthcare system	2 (10.5)	[14,17]
	Outpatient clinic (e.g., internal medicine clinic, primary care, ambulatory oncology)	10 (52.6)	[4,15,16,17,18,19,20,22,23,26]
	Not specified	1 (5.3)	[30]

**Table 3:** Summary of the evidence of the included studies.

Study	Sample Size and Data Source	Study Design	Outcome Measures	Findings
Adler-Milstein et al., 2020 [13] ^†^ **Study Purpose:** this study explored how acute-care hospital electronic health records (EHRs) supported documentation of care for older adult patients using the 4Ms Framework.	**Setting and** **Country:** acute-care hospital in the USA. **Sample Population:** N = 479 hospitals (60.1% response rate). **Data Source:** national random sample of 797 acute-care hospitals from 2018–2019 in the USA (the age distribution of the sample was not provided).	Cross-sectional quantitative survey study. **Intervention:** not applicable.	An online survey was used regarding hospital EHR functions to support age-friendly care using the 4Ms Framework (30 questions). This study focused on survey questions related to how the hospital had implemented structured EHR documentation to record the metrics associated with the 4Ms Framework, including patient care goals, medications, challenges around mentation, and mobility.	Among the 479 completed surveys, 64% of the hospitals had implemented structured documentation in the EHR using the 4Ms Framework in at least 1 unit. Note that 41.5% had implemented the structured documentation across all hospital units. The most frequent adoption was for medication (91.3% in at least 1 unit) and caregiver information, and the least frequent adoption was for mentation (70.3% in at least 1 unit). There was low adoption of (1) training for older adults and family caregivers on the patient portal, (2) an electronic medical record portal for long-term care facilities, and (3) being able to electronically send information to long-term care facilities.
Adler-Milstein et al., 2023 [4] ^†^ **Study Purpose:** this qualitative study conducted interviews to assess the implementation of the 4Ms Framework in early-adopter health systems. Approaches to and experiences with 4Ms Framework implementation (e.g., facilitators and barriers) were explored.	**Setting and Country:** academic and non-academic inpatient and outpatient clinical settings in the USA. **Sample Population:** N = 29 stakeholders at 3 health system sites (21 from an inpatient setting and 8 from an outpatient setting). Among these 29 stakeholders, 22 were frontline clinicians (e.g., physicians, nurses, pharmacists, physical therapists, and social workers), and 7 were those in administrative or leadership positions. **Data Source:** semi-structured interviews from stakeholders in three healthcare systems: Anne Arundel Medical Center, Maryland; University of Utah; and University of California, San Francisco.	A cross-sectional qualitative study using a semi-structured questionnaire. **Intervention:** not applicable.	Not applicable.	The 4Ms Framework offered a compelling conceptual framework for advancing age-friendly care. However, implementation was complex and fragmented. Each health system took varied implementation approaches with a different implementation order for each of the 4Ms. None of the sites implemented all components of the 4Ms at one time due to the number of activities and disciplines involved. The common strategies that facilitated the 4Ms implementation success and supported frontline culture change were: (1) continuous communication from leadership promoting the 4Ms as a priority, (2) engagement across multiple disciplines, (3) adopting EHR templates for understanding clinical workflows, promoting adherence to standardized 4Ms process and reporting, (4) the use of peer coaching and clinical champions who attended unit meetings and clinical huddles, providing hands-on support and clinical education, and (5) incorporating compliance incentives. The researchers identified three common barriers to the implementation of the 4Ms: (1) physician disengagement due to the perception of the 4Ms as not being their work responsibility, (2) siloed implementation efforts across settings within a health system, which limited synergies and scaling of the 4Ms Framework, and (3) difficulty in knowing how to implement “What Matters” meaningfully.
Berish et al., 2023 [26] ^†^ **Study Purpose:** this intervention study examined the impact of using a telementoring educational program for clinician training about the 4Ms Framework on changes in adopting the 4Ms Framework at 3 federally qualified health centers within the Primary Health Network (PHN).	**Setting and Country:** Primary care in the USA. **Sample Population:** N = 397 patients aged 65 years and older, who received care from one of the 3 pilot sites; N = 6527 patients aged 65 years and older, who received care from one of the 18 comparison sites. **Data Source:** electronic medical record.	Prospective/intervention study with a comparison group. **Intervention:** A telementoring educational program for clinician training about the 4Ms Framework was implemented.	Annual wellness visit (AWV) completion. Advance care. Planning documentation. Identification and documentation of the caregiver. High-risk medication elimination. Opioid misuse mitigation. Dementia screening and monitoring. Fall risk management. Setting mobility goal.	All nine outcomes were significantly increased by 4–43% between the baseline and follow-up measurements among the older adults who received care from one of the three pilot sites.
Breda et al., 2023 [27] ^†^ **Study Purpose:** this quantitative study determined the outcomes of adopting the 4Ms Framework in an integrated inpatient and outpatient program specifically for geriatric fracture patients.	**Setting and Country:** inpatient acute care hospital in the USA. **Sample Population:** N = 1598 patients aged 65 years and older with fractures; 746 managed by a Geriatric Fracture Program (GFP) physician, and 852 managed by non-GFP physicians. **Data Source:** electronic medical record.	A quantitative retrospective survey using clinical data. **Intervention:** not applicable.	Time to surgery (hours). Length of stay (days). Vizient length of stay index. Total direct cost (US dollars). 30-day readmissions. 30-day mortality. 90-day mortality.	Geriatric Fracture Program patients had significantly lower direct costs, length of stay, and length of stay index. However, there was no significant difference in time to surgery. 23% of GFP patients had a postdischarge visit within six months or less.
Casey et al., 2020 [15] ^†^ **Study Purpose:** this pre- and posttest survey study assessed changes in primary care physicians’ knowledge, confidence, and clinical practice for evaluating and managing fall risks.	**Setting and Country:** professional development for primary care physicians in the USA. **Sample Population:** N = 6 primary care physicians from different clinics representing rural and urban settings. **Data Source:** pre- and postsurveys and patient data.	Quality improvement project/mixed methods survey study. **Intervention:** A 1-week, in-depth, skills-based geriatric educational session (4 full days) focused on the 4Ms Framework component of mobility (part of a 4-week geriatric mini fellowship) was implemented.	11-item multiple-choice questionnaire. 4-item confidence questionnaire. Qualitative comments from evaluations following the educational session. Patient data 12 months before and after the fall risk training to evaluate primary care physicians’ adoption of any of the fall risk management components.	After the 4-week geriatric mini fellowship training, primary care providers were 1.7 times more likely to screen for fall risk and 3.6 times more likely to discuss fall risk. Primary care providers were also 5.8 times more likely to assess patients aged 65 and older for orthostatic blood pressure. Regarding high-risk older adult patients, participating primary care providers were 4.1 times more likely to discuss fall risk and 6.3 times more likely to assess orthostatic blood pressure than their peers who did not receive the education.
Dolansky et al., 2021 [18] ^†^ **Study Purpose:** this paper described the preimplementation phase to integrate the 4Ms Framework in MinuteClinics of CVS Health. MinuteClinics provide both in-person and virtual care visits.	**Setting and Country:** primary care clinics (Minute Clinics of CVS Health in the USA). **Sample Population:** N = 1100 clinics across 33 states and the District of Columbia. These clinics were staffed by more than 3000 nurse practitioners and physician assistants. **Data Source:** stakeholders (i.e., patients, healthcare providers, clinic managers, educators, informatics and communication staff, and implementation consultants) using observations, surveys, interviews, focus groups, organizational readiness assessments, stakeholder assessments, and workflow mapping.	Quality improvement study with a focus on the preimplementation stage of the 4Ms Framework. **Intervention:** not applicable.	NA	During the 15-month preimplementation period, the researchers identified potential barriers, facilitators, and opportunities for implementation of the 4Ms Framework. The authors developed the Age-Friendly Health Systems ambulatory care continuum logic model to realize opportunities to implement the 4Ms Framework within MinuteClinics.
Gettel et al., 2022 [24] * Study purpose: this qualitative study conducted dyadic semi-structured interviews of cognitively intact older adults and treating clinicians in the emergency department (ED) setting to describe the concerns and desired outcomes of seeking ED care. The “What Matters” conversation guide based on the 4Ms Framework was used to achieve the study aim.	**Setting and Country:** emergency departments in the USA. **Sample Population:** N = 46 older adults with intact cognitive abilities aged 70 years and older (mean age = 87 years; 27 were female), English-speaking, ability to answer questions themselves without assistance from family caregivers, and low acuity at the ED triage/an emergency severity index score of 3–5; N = 46 matched treating physicians (74%) or non-physician practitioners (26%). **Data Source:** digitally recorded interviews during ED visits in a community hospital and Level II trauma center within the same health system.	Cross-sectional qualitative study. **Intervention:** not applicable.	Concerns and desired outcomes of seeking EDE care. Feasibility of incorporating the “What Matters” questions within the ED clinical practice.	The three most common chief complaints were falls/musculoskeletal concerns (35%), weakness, fatigue, or dizziness (24%), and cardiopulmonary issues (22%). The four most common body systems associated with ED diagnoses were musculoskeletal (26%), cardiopulmonary (18%), infection (15%), and electrolyte disturbance or metabolic (15%). Twenty-six (57%) older adult participants were admitted for acute hospitalization. Patients and their ED treating clinicians shared similar concerns and desired outcomes. Clinicians perceived that older adult patients were worried about identification of symptom cause as the primary concern.
Greenberg et al., 2022 [22] ^†^ **Study Purpose:** this study described the quality improvement process of developing and pilot-testing clinician/staff educational materials for integrating the 4Ms Framework into CVS Health MinuteClinics.	**Setting and Country:** primary care clinics (1200 CVS Health MinuteClinics in 35 states and the District of Columbia in the USA). **Sample Population:** healthcare providers and educators in 1200 MinuteClinics. No inclusion or exclusion criteria. As for the patients served from 2018–2019, about 15% of the MinuteClinic visits were for older adults aged 65 years and older. **Data Source:** surveys, focus groups, interviews, and site visits.	Quality improvement study with a focus on developing educational materials for adopting the 4Ms Framework in MinuteClinics. **Intervention:** not applicable.	NA.	The quality improvement process identified educational gaps related to the 4Ms Framework and age-friendly care. The feedback from the healthcare providers in the MinuteClinics informed the final versions of the educational materials (i.e., the orientation module, video vignettes, and monthly grand rounds). Having time to complete the educational materials and engage in the monthly grand round remains a barrier.
Guth et al., 2020 [16] ^‡^ **Study Purpose:** this case study described implementing the 4Ms Framework in an interprofessional outpatient clinic focusing on high-risk medication use and deprescribing to prevent high-cost outcomes (e.g., emergency department visits).	**Setting and Country:** interprofessional outpatient clinic in the USA. **Sample Population:** N = 67 new adult patients during the pre-intervention period (age range: not specified); N = 55 new adult patients during the intervention period (age range: not specified). **Data Source:** electronic medical records.	Prospective quality improvement study. **Intervention:** the 4-Ms-centric intervention components included documentation of initial interdisciplinary comprehensive evaluation, risk assignment, high-risk rounds among the primary care team, and identified high-risk medications.	Improvement in medication adherence and simplification of medication regimens. Reduction in adverse drug reactions. Cost savings from medication management. Reduction in hospitalizations and re-admissions and emergency department visits.	During the intervention period, 69% of new patients received a mobility screening (an increase from 55% during the preintervention period), 85% had a mental examination (increase from 82%), 85% consulted with their pharmacist to manage their medications (no change from preintervention period), and 69% had “What Matters” to the patients addressed (decrease from 85%, mainly due to failing to upload intake notes to electronic medical records). The 4Ms intervention resulted in an improvement in medication adherence, simplification of medication regimens, and a reduction in hospitalizations and readmissions.
Kuntz et al., 2023 [28] ^†^ **Study Purpose:** this quality improvement project sought to describe the process of implementing an intervention to increase the use of the 4Ms Framework within an inpatient consultative geriatric care practice.	**Setting and Country:** inpatient consultative geriatric care in the USA. **Sample Population:** N = 421 veterans aged 65 years and older were evaluated by the Elder Veteran Program (EVP); N = 2265 veterans aged 65 years and older admitted to the hospital. **Data Source:** electronic medical record.	Retrospective/quantitative quality improvement study. **Intervention:** the Plan–Do–Study–Act process was used to increase the completeness of adoption of the 4Ms Framework related to documentation and to modify templates and workflow to enhance the quality of consultation and documentation.	Percentage of documentation notes with medication screen. Percentage of documentation notes with delirium assessment and interpretation. Percentage of documentation notes making comprehensive delirium risk recommendations. Percentage of patients with Morse Fall Score reported. Percentage of patients with the use of assistive devices. Percentage of patients with “What Matters” documented and determined.	Medication documentation improved from 34.0% to 82.2%. Mentation documentation of mental status improved from 62.3% to 94.4%, and implementation of delirium reduction measures improved from 28.8% to 99.3%. Before implementation of the intervention, 100% of the cases already included documentation of the Morse Fall Scale and recommendations for mobility safety and assistive devices; no changes after introducing the intervention. ”What Matters” documentation improved from 56.6% to 89.9%.
Lesser et al., 2022 [23] ^†^ **Study Purpose:** this survey examined family and internal medicine specialists’ attitudes, knowledge, and practice related to the 4Ms Framework in their primary care clinics.	**Setting and Country:** Primary care clinics in the USA. **Sample Population:** N = 575 physicians; N = 613 nurse practitioners; N = 496 physician assistants. Participants must be family medicine or internal medicine specialists who see at least 25 patients aged 65 years and older in an average month and self-identify as White, Black, Hispanic origin, or Asian. **Data source:** USA-based healthcare providers were randomly identified from the Medscape database; response rate to the online survey was 0.9%.	Cross-sectional quantitative survey study. **Intervention:** not applicable.	Online survey responses.	Over 90% of the clinicians stated that older patients needed a different approach than younger patients, and 50% said they “always” considered the patient’s age when providing care. About 60% of the clinicians were either not currently using the 4Ms Framework of Age-Friendly Health Systems in their practice settings (40%) or were unaware whether their practice settings were adopting the 4Ms Framework (20%). The healthcare team’s lack of familiarity with the 4Ms and lack of time during the visits were two common barriers for clinicians and their teams to address the 4Ms. Physicians and physician assistants found the mentation component in the 4Ms Framework to be the most challenging one to address with older adults. Nurse practitioners found the medication component the most challenging one to address. The mobility component was the least challenging for all these primary care clinicians. About 30% of the clinicians were not asking their older patients “What Matters” for alignment with their care plans.
Lundy et al., 2021 [17] ^†^ **Study Purpose:** this quality improvement study described adopting the 4Ms Framework to support a geriatric syndrome screening using the Rapid Geriatric Assessment and an intervention program at a rural healthcare system’s primary care clinic.	**Setting and Country:** rural healthcare system’s primary care clinic in the USA. **Sample Population:** N = 1326 older adults aged 65 years and older coming to the outpatient clinic in a rural primary healthcare system, Perry County, Missouri, USA. Among 141 older adults who completed the cognitive stimulation therapy and the exercise program, 21 completed all follow-up evaluations. Among 88 older adults who completed the exercise program, 16 completed all follow-up evaluations. **Data Source:** patients in Perry County, Missouri.	A quantitative retrospective survey using clinical data. **Intervention:** based on assessment results, 3-month exercise therapy for muscle strength and function and 7-week cognitive stimulation therapy for cognitive dysfunction were implemented.	Five Times Sit to Stand (FTSS), Timed Up and Go (TUG), Cornell Scale for Depression in Dementia (CSDD), Saint Louis University Mental Status Examination (SLUMS), Quality of Life in Alzheimer’s Disease (QoL-AD) measures.	Both the exercise program and the cognitive stimulation therapy improved outcomes. Individuals who received exercise therapy had an improvement in TUG and FTSS scores at 3 months and 12 to 24 months. For individuals who received cognitive stimulation therapy, the SLUMS, QoL-AD, and CSDD improved at 7 weeks and 6 to 12 months. This quality improvement initiative that introduced a screening program for geriatric syndromes is feasible.
Lynch et al., 2021 [19] ^†^ **Study Purpose:** this study used an online survey to explore the perceptions of ambulatory oncology leaders regarding the current practice and readiness of cancer programs to provide age-friendly care and implement the 4Ms Framework.	**Setting and Country:** ambulatory oncology care in the USA. **Sample Population:** N = 81 (i.e., nurses, physicians, and cancer service administrators). **Data Source:** study participants were recruited through professional organization memberships.	Mixed methods/cross-sectional survey study. **Intervention:** not applicable.	A 24-item survey composed of multiple choice and open-ended questions that were based on the elements of the 4Ms Framework.	Sixty-seven percent of participants responded that their facility plans to achieve age-friendly cancer care within 5 years. Seventy-seven percent responded that their employees do not have specialized training or education regarding the care of older adults. Eleven percent indicated that interventions to address “What Matters” had been implemented. Fourteen percent indicated that interventions to address mentation were implemented. Twenty-seven percent indicated that interventions to address medication had been implemented. Thirty-two percent indicated interventions to address mobility were implemented. Twelve percent indicated that their facility’s leadership was fully engaged in moving towards age-friendly cancer care. Twenty-six percent said that their leadership was unaware or not engaged yet. Thirty-six percent responded that their leadership was aware and only partially involved in moving towards age-friendly cancer care and was focused on other priorities.
McQuownet al., 2023 [29] ^†^ **Study Purpose:** this clinical pilot study determined the feasibility of implementing a postemergency department follow-up program that combined home and telehealth visits for older veterans after emergency department discharges.	**Setting and Country:** 6 U.S. Department of Veterans Affairs geriatric emergency departments (EDs). **Sample Population:** N = 6 U.S. Department of Veterans Affairs Eds; N = 56 telephone visits; N = 247 home visits, 3 completed by a geriatrician and 244 completed by an ED provider. **Data Source:** survey used to assess existing geriatric ED processes and Veterans Affairs Corporate Data Warehouse data.	Feasibility pilot intervention study. **Intervention:** the pilot intervention is Supporting Community Outpatient, Urgent Care and Telehealth Services (SCOUTS). Six U.S. Department of Veterans Affairs EDs identified high-risk older adult patients during an ED visit. After ED discharge, intermediate care technicians (i.e., formal military medics) performed follow-up telephone or home visits. During follow-up home visits, intermediate care technicians identified “What Matters”, performed geriatric screens and home risk assessment, and provided care coordination using the 4Ms Framework. Home visits were assisted with video telehealth check-ins with ED providers.	Feasibility of SCOUTS in assisting with the ED to home transitions of care, identifying unmet needs, and increasing access to healthcare services using telehealth technologies.	Among the 247 home visits, the intermediate care technicians identified 99 unmet care needs and 44 modifiable home fall risks. Using the SCOUTS to promote 4Ms of Age-Friendly Health Systems is feasible through assisted telehealth. Combining telehealth and home visits after emergency department visits could help Veterans Affairs ED clinicians address patients’ “What Matters” and identify unmet care needs.
Morgan et al., 2022 [20] ^†^ **Study Purpose:** this study examined the relationship of five health equity variables (sex, race, ethnicity, preferred language, and electronic patient portal account activation) against the 4Ms Framework for patients in an academic internal medicine clinic.	**Setting and Country:** academic internal medicine clinic in the USA. **Sample Population:** N = 3370 patients aged 65 years and older (56.9% were females, 86.2% were White, 90.8% were non-Hispanic, 96.8% identified English as the preferred language, and 93% had activated their electronic patient portal account). **Data Source:** Oregon Health & Science University’s Internal Medicine and Geriatrics Clinic electronic medical records for patients who completed visits between April 2020 and April 2021.	Retrospective quantitative study. **Intervention:** not applicable.	The “What Matters” metric was met with documentation discussing prognosis and end of life care. The “Medication” metric was met if no high-risk medications were active on an individual patient’s current medication list. The “Mobility” metric was met if a fall risk screening tool was completed within the last year. The “Mentation” metric was met if depression and cognition screenings were completed within the last year.	Five hundred and seventeen (15.3%) patients received care, including all 4Ms. Advance care planning discussions occurred more often with females than males and with English speakers than non-English speakers. Females were more likely to use at least one high-risk medication than males. Patients with an activated electronic patient portal account were more likely to use high-risk medications than the ones with an inactive account. Individuals with an active electronic patient portal account were more likely to have cognitive screening than the ones without an active account.
Severance et al., 2022 [25] ^Ø^ **Study Purpose:** this prospective study examined the outcomes of a fall prevention training program (“A Matter of Balance”, a community-based health promotion intervention) using the 4Ms Framework of Age-Friendly Health Systems. This training program involved a partnership among an academic institution, emergency management services, community organizations, and government agencies.	**Setting and Country:** independent residential communities, faith-based organizations, healthcare organizations, activity centers, and libraries in the USA. **Sample Population:** N = 141 participants completed the baseline and postintervention surveys; targeted age range: no age limit to participate in the intervention (mean age: 76.37 years, range: 54–94); 80% were female; 86.7% were White, 8.6% Black, 0.5% Asian, 1.1% American Indian or Alaska Native, 92.6% not Hispanic or Latino; 97.3% spoke English as the primary language, 1.1% spoke Spanish). **Data Source:** Participants were recruited through community-based organizations in north Texas.	Prospective quality improvement study. **Intervention:** “A Matter of Balance” training program for fall prevention addressing the 4Ms of Age-Friendly Health Systems was tested. The intervention was delivered over 12 months and included eight small group sessions on identifying and controlling modifiable fall risk factors including home safety evaluation, physical activity, and practicing assertiveness. Each session included goal setting; strength, coordination, balance exercises; group discussion; and problem-solving.	The 5-Item Falls Efficacy Scale Health-Related Quality of Life Survey	The intervention resulted in a statistically significant improvement in fall efficacy for older adults. Thus, there was no statistically significant change in the self-assessment of health-related quality of life levels.
Shen et al., 2022 [21] ^†^ **Study Purpose:** this quality improvement study evaluated the intervention of establishing a hospital medicine–orthopedics co-management program to transform an orthopedic unit into an age-friendly one in a hospital without onsite geriatricians and a dedicated geriatrics unit.	**Setting and Country:** acute-care hospital’s inpatient orthopedic unit in the USA. **Sample Population:** hospitalized adults aged 60 years and older with fragility fracture of the native proximal femur. N = 436 patients for delirium; N = 479 patients for the weight-bearing analysis. **Data Source:** orthopedic department billing records for patients 60 years and older with CPT codes that applied to surgical repair of proximal femur fractures.	Retrospective, non-randomized, quality improvement study. **Intervention:** a hospital medicine–orthopedics co-management model for a geriatric fracture center was developed to support the four principles in the 4Ms Framework with 12 components: two were tested: incorporating mobility specialists (licensed practice nurses) to improve early mobilization on the orthopedic unit and delirium reduction strategies (i.e., changing blood draw times to avoid sleep disturbance).	Frequency of weight-bearing on postoperative day 1. Frequency of delirium.	The age-friendly intervention reduced delirium frequency by 26% for patients on the intervention unit compared to 35% on other units (not statistically significant, *p* = 0.055). There was an 84% frequency of day 1 postoperative weight-bearing for patients in the intervention unit versus 72% in other units (statistically significant, *p* = 0.003). There was no change in the median length of stay in the intervention unit.
Wang et al., 2023 [30] ^‡^ **Study Purpose:** this scoping review used the 4Ms principles in the 4Ms Framework to evaluate the age-friendliness of deprescribing trials, specifically in intervention design and outcome measures. The goal was to identify gaps in the existing literature, focusing on learning how often each of the 4Ms components was considered in the intervention design and outcome measurement processes.	**Setting and Country:** not limited to any specific clinical settings; 34 studies conducted in European countries; 3 studies conducted in the USA. **Sample Population:** N = 37 included studies; 3 were conducted in the United States, and most were conducted in European countries, Canada, and some Asian countries. Patients aged 65 years or older. Eleven of the studies were conducted in nursing homes, eleven in hospitals, one in an outpatient ambulatory care setting, and fourteen in the community through primary care (n = 8), community pharmacies (n = 3), assisted living settings (n = 2), and patient’s home (n = 1). **Data Source:** published literature from MEDLINE, EMBASE, CINAHL, the Cochrane Central Register of Controlled Trials, Web of Science, and ProQuest databases and snowball method from the reference lists of selected articles and published reviews.	Scoping review. **Intervention:** not applicable.	Number of trials including any of the 4Ms components of the medication, mentation, mobility, and “What Matters” in the intervention designs and outcome assessments.	All 37 studies regarding deprescribing intervention trials considered medication. Eight trials considered mentation, two trials considered mobility, and six trials considered “What Matters most”. Thirty-three trials assessed medication outcomes and thirteen assessed mobility outcomes. Ten assessed mentation outcomes. Thus, none of the included studies assessed “What Matters most” outcomes. Mentation, mobility, and “What Matters most” were varyingly considered in existing deprescribing trials.
Winterton et al., 2021 [14] ^†^ **Study purpose:** this integrative review identified the core elements or interventions that facilitated implementing the 4Ms Framework for older adults within rural Australian health systems.	**Setting and Country:** rural health systems in Australia. **Sample Population:** adults aged 65 years and older in rural Australia. **Data Source:** MEDLINE, CINAHL, Scopus, Embase, PsycINFO, Web of Science, Informit Rural Health, PubMed, Joanna Briggs Institute, and Cochrane Database of Systematic Reviews databases.	Integrative review study. **Intervention:** not applicable.	Twenty-four peer-reviewed journal articles were included in the synthesis.	The evidence indicates that the 4Ms Framework was feasible in the rural Australian context. There were more reviewed studies on mobility and mentation, with fewer reviewed studies relating to medications and ”What Matters”. Over one-third of the reviewed studies solely focused on mobility; 4 focused on fall prevention as the intervention.

^†^ = Addressing the overall 4Ms Framework of Age-Friendly Health Systems; * = Addressing the “What Matters” component only; ^‡^ = Addressing the “Medication” component only; ^Ø^ = Addressing the “Mobility” component only.

**Table 4:** Facilitator themes on implementing the 4Ms Framework of Age-Friendly Health Systems.

Facilitator Theme	Subthemes
Frequency of patient participation in age-friendly care [20]	Advance care planning discussion [20] Preventing high-risk medications [20] Cognitive screening [20]
Aligning the health system’s mission with the 4Ms Framework [4,18]	Health system climate readiness [18] Alignment with clinics’/health systems’ purpose in improving health [18] Leadership, clinicians, and staff buy-in [4,18]
Infrastructurereadiness to embrace the 4MsFramework [4,13,15,16,18,25,26,27,28]	The 4Ms Framework implementation approaches [16,25,28] Training and professional development opportunities [4,15,18,26] Informatics integration [4,13,18] Communication channels among healthcare delivery team members [18] Interprofessional communication and collaboration regarding patient care plan [16,27] Quality monitoring and evaluation processes [4,18]
Embedding the 4Ms Framework intoroutine clinical practice with clinical pathways and designatedpersonnel [14,16,17,18,21,23,24,25,27,28,29]	Adopting geriatric syndrome screening before introducing interventions [17,27] Alignment between the entire 4Ms Framework or part of the 4Ms Framework and clinical practice [14,16] Alignment between the 4Ms Framework component of “What Matters?” and patients’ interests in improving health [18,24,27,28,29] Alignment between the 4Ms Framework component of mentation and clinical practice [21,27] Alignment between the 4Ms Framework component of medication and clinical practice [16,27,28] Alignment between the 4Ms Framework component of mobility and clinical practice [21,23,25,27]

**Table 5:** Barrier themes on implementing the 4Ms Framework of Age-Friendly Health Systems.

Barrier Theme	Subthemes
Patients unable to actively participate in age-friendly care [18,20,25]	Challenges in communicating the benefits of the 4Ms Framework with older adults [18] Advance care planning discussion [20] Preventing high-risk medications [20] Cognitive screening [20] Patients being unable to participate [25]
Lack of infrastructure readiness to embrace the 4Ms Framework in clinical practice [13,16,19,29]	Lack of leadership engagement in the 4Ms Framework implementation [19] Lack of structured documentation using the 4Ms Framework in the electronic medical records [13,29] Lack of infrastructure for training and communications [13,16,19]
Lack of clinicians’buy-in [4,13,18,22,23,25,28,30]	Clinicians’ concerns with adding extra burden and steps during clinic visits [18,23] Lack of time to learn about the 4Ms Framework [22,23] Limited time to implement the 4Ms Framework [13,18,23,25] Lack of full buy-in from clinicians and health systems in eliciting older adults’ goals and values (i.e., “What Matters?”) [28] Limited published literature on implementation of the 4Ms Framework [30] Siloed implementation efforts across settings within a health system leading to limited synergies and scaling of the 4Ms Framework [4]
Challenges in incorporating the 4Ms components in clinical practice [4,19,23,25,29]	Lack of a formalized pathway to identify “What Matters” [4,23] Lack of interventions to address the “What Matters”, mentation, medication, and mobility components of the 4Ms Framework during clinic visits [19,23] Physicians and physician assistants have difficulties with addressing mentation during clinic visits [23] Nurse practitioners have challenges in addressing medication concerns during clinic visits [23] Limited numbers of staff to support 4 M Framework implementation [25]
Lack of clinician awareness [4,18,23]	Lack of knowledge of the 4Ms Framework of Age-Friendly Health Systems [18,23] Lack of knowledge of community resources [18] Lack of clarity regarding implementation of the 4Ms [4,18]

**Table 6:** Summary of the identified facilitator and barrier themes on implementing the 4Ms Framework.

		n (%)	Article Citations
Facilitator themes	Frequency of patient participation in age-friendly care	1 (5.3)	[20]
	Aligning the health system’s mission with the 4Ms Framework	2 (10.5)	[4,18]
	Infrastructure readiness to embrace the 4Ms Framework	9 (47.4)	[4,13,15,16,18,25,26,27,28]
	Embedding the 4Ms Framework into routine clinical pathways and designated personnel responsibilities	11 (57.9)	[14,16,17,18,21,23,24,25,27,28,29]
Barrier themes	Patients unable to actively participate in age-friendly care	3 (15.8)	[18,20,25]
	Lack of infrastructure readiness to embrace the 4Ms Framework in clinical practice	4 (21.1)	[13,16,19,29]
	Lack of clinicians’ buy-in	8 (42.1)	[4,13,18,22,23,25,28,30]
	Challenges in incorporating the 4Ms components in clinical practice	4 (21.1)	[4,19,23,25,29]
	Lack of clinician awareness	3 (15.8)	[4,18,23]

## Data Availability

No new data were created. Readers may request the corresponding author’s summary information in the tables via email.

## Supplementary Materials

The following supporting information can be downloaded at: https://www.mdpi.com/article/10.3390/nursrep14020070/s1, Table S1: Critical appraisal of the included analytical cross-sectional studies using the Joanna Briggs Institute critical appraisal tools for study designs; Table S2: Critical appraisal of the included qualitative research using the Joanna Briggs Institute critical appraisal tools for study designs; Table S3: Critical appraisal of the included systematic reviews and research syntheses using the Joanna Briggs Institute critical appraisal tools for study designs; Table S4: Critical appraisal of the included studies reporting prevalence data using the Joanna Briggs Institute critical appraisal tools for study designs; Table S5: Critical appraisal of the included studies reporting prevalence data using the Joanna Briggs Institute critical appraisal tools for study designs.

## Public Involvement Statement

No public involvement in any aspect of this research.

## Guidelines and Standards Statement

This manuscript was drafted according to the guidelines of the Preferred Reporting Items for Systematic Reviews and Meta-Analyses Extension for Scoping Reviews (PRISMA-SCR) [8].
