# Supplementary material for: Facilitators and Barriers to Implementing the 4Ms Framework of Age-Friendly Health Systems: A Scoping Review
Source: Nurs Rep. 2024 Apr 15;14(2):913–30. doi: 10.3390/nursrep14020070 (PMC11036203; doi:10.3390/nursrep14020070)
Supplement: Supplementary file 1 [file nursrep-14-00070-s001.zip › nursrep-2905189-supplementary.pdf]

Supplemental Table S1. Critical appraisal of the included analytical cross-sectional studies using the Joanna Briggs Institute critical appraisal tools for study designs.

	**Analytical cross-sectional study**	**Adler-Minstein et al., 2020**[13]	**Lesser et al., 2022**[23]	**Dolansky et al., 2021**[18]	**Morgan et al., 2022**[20]	**Lynch et al., 2021**[19]
1	Were the criteria for inclusion in the sample clearly defined?	Y	Y	Y	Y	Y
2	Were the study subjects and the setting described in detail?	Y	Y	Y	Y	Y
3	Was the exposure measured in a valid and reliable way?	Y	Y	U	Y	Y
4	Were objective, standard criteria used for measurement of the condition?	Y	Y	Y	Y	Y
5	Were confounding factors identified?	NA	NA	U	NA	NA
6	Were strategies to deal with confounding factors stated?	NA	NA	U	NA	NA
7	Were the outcomes measured in a valid and reliable way?	Y	Y	Y	Y	NA
8	Was appropriate statistical analysis used?	Y	Y	U	Y	Y

Key: Y= Yes; N=No; U=Unclear; NA=Not applicable; EHR = Electronic Health Record.

Supplemental Table S2. Critical appraisal of the included qualitative research using the Joanna Briggs Institute critical appraisal tools for study designs.

	**Qualitative research**	**Adler-Milstein et al., 2023[4]**	**Gettel et al., 2022[24]**	**Greenberg et al., 2022[22]**
1	Is there congruity between the stated philosophical perspective and the research methodology?	Y	Y	Y
2	Is there congruity between the research methodology and the research question or objectives?	Y	Y	Y
3	Is there congruity between the research methodology and the methods used to collect data?	Y	Y	Y
4	Is there congruity between the research methodology and the representation and analysis of data?	Y	Y	Y
5	Is there congruity between the research methodology and the interpretation of results?	Y	Y	Y
6	Is there a statement locating the researcher culturally or theoretically?	Y	Y	NA
7	Is the influence of the researcher on the research, and vice-versa, addressed?	N	Y	Y
8	Are participants, and their voices, adequately represented?	Y	Y	Y
9	Is the research ethical according to current criteria or, for recent studies, and is there evidence of ethical approval by an appropriate body?	Y	Y	Y
10	Do the conclusions drawn in the research report flow from the analysis, or interpretation, of the data?	Y	Y	Y

Key: Y= Yes; N=No; U=Unclear; NA=Not applicable.

Supplemental Table S3. Critical appraisal of the included systematic reviews and research syntheses using the Joanna Briggs Institute critical appraisal tools for study designs.

	Systematic reviews and research syntheses	Wang et al., 2023[30]	Winterton et al., 2021[14]
1	Is the review question clearly and explicitly stated?	Y	Y
2	Were the inclusion criteria appropriate for the review question?	Y	Y
3	Was the search strategy appropriate?	Y	Y
4	Were the sources and resources used to search for studies adequate?	Y	Y
5	Were the criteria for appraising studies appropriate?	Y	Y
6	Was critical appraisal conducted by two or more reviewers independently?	Y	Y
7	Were there methods to minimize errors in data extraction?	Y	Y
8	Were the methods used to combine studies appropriate?	Y	Y
9	Was the likelihood of publication bias assessed?	Y	Y
10	Were recommendations for policy and/or practice supported by the reported data?	Y	Y
11	Were the specific directives for new research appropriate?	Y	Y

Key: Y= Yes; N=No; U=Unclear; NA=Not applicable.

Supplemental Table S4. Critical appraisal of the included studies reporting prevalence data using the Joanna Briggs Institute critical appraisal tools for study designs.

	**Studies reporting prevalence data**	**Lundy et al., 2021[17]**
1	Was the sample frame appropriate to address the target population?	Y
2	Were study participants sampled in an appropriate way?	Y
3	Was the sample size adequate?	Y
4	Were the study subjects and the setting described in detail?	Y
5	Was the data analysis conducted with sufficient coverage of the identified sample?	Y
6	Were valid methods used for the identification of the condition?	Y
7	Was the condition measured in a standard, reliable way for all participants?	Y
8	Was there appropriate statistical analysis?	Y
9	Was the response rate adequate, and if not, was the low response rate managed appropriately?	

Key: Y= Yes; N=No; U=Unclear; NA=Not applicable.

Supplemental Table S5. Critical appraisal of the included studies reporting prevalence data using the Joanna Briggs Institute critical appraisal tools for study designs.

	Quasi-experimental studies	Berish et al., 2023[26]	Breda et al., 2023[27]	Casey et al., 2020[15]	Guth et al., 2020[16]	Kuntz et al., 2023[28]	McQuown et al., 2023[29]	Severance et al., 2022[25]	Shen et al., 2022[21]
1	Is it clear in the study what is the 'cause' and what is the 'effect' (i.e., there is no confusion about which variable comes first)?	Y	Y	Y	Y	Y	Y	Y	Y
2	Were the participants included in any comparisons similar?	Y	Y	Y	Y	Y	Y	Y	Y
3	Were the participants included in any comparisons receiving similar treatment/care, other than the exposure or intervention of interest?	Y	Y	Y	Y	Y	Y	Y	Y
4	Was there a control group?	Y	Y	Y: a pre–postintervention design	Y: a pre–postintervention design	Y: a pre–postintervention design	Y: a pre–postintervention design	Y: targeted areas vs. non-targeted areas	Y
5	Were there multiple measurements of the outcome both pre and post the intervention/exposure?	Y	Y	Y	Y	Y	Y	Y	Y
6	Was follow up complete and if not, were differences between groups in terms of their follow up adequately described and analyzed?	Y	Y	Y	Y	Y	Y	Y	Y
7	Were the outcomes of participants included in any comparisons measured in the same way?	Y	Y	Y	Y	Y	Y	Y	Y
8	Were outcomes measured in a reliable way?	Y	Y	Y	Y	Y	Y	Y	Y
9	Was appropriate statistical analysis used?	Y	Y	Y	Y	Y	Y	Y	Y

Key: Y= Yes; N=No; U=Unclear; NA=Not applicable.
